# Investigating population dynamics from parentage analysis in the highly endangered fan mussel *Pinna nobilis*

**DOI:** 10.1002/ece3.8482

**Published:** 2022-01-25

**Authors:** Claire Peyran, Emilie Boissin, Titouan Morage, Elisabet Nebot‐Colomer, Guillaume Iwankow, Serge Planes

**Affiliations:** ^1^ EPHE – UPVD – CNRS USR 3278 CRIOBE PSL Research University Perpignan France; ^2^ Laboratoire d'Excellence «CORAIL» Perpignan France; ^3^ Instituto Español de Oceanografía (IEO, CSIC), Centro Oceanográfico de Baleares Palma de Mallorca Spain

**Keywords:** conservation, external spawner, larval dispersal, parentage analysis, recruitment, self‐recruitment

## Abstract

Understanding dispersal patterns is a major focus for conservation biology as it influences local survival and resilience in case of local disturbance, particularly for sessile species. Dispersal can be assessed through parentage analyses by estimating family structure and self‐recruitment. This study documents the family structure of a pelagic spawner, *Pinna nobilis*, which is facing a major crisis that threatens its survival as most of its populations have been decimated by a parasite, *Haplosporidium pinnae*. In this context, we focused on a single population (Peyrefite, Banyuls‐sur‐mer, France) where 640 individuals were sampled in 2011, 2015, and 2018 and genotyped for 22 microsatellite markers. Genetic diversity was high and homogeneous among years, with mean allele numbers ranging between 13.6 and 14.8 and observed heterozygosities (*H*_o_) between 0.7121 and 0.7331. Low, but significant, genetic differentiations were found between 2011–2015 and 2015–2018. A parentage analysis described 11 clusters, including one prevailing, and revealed that 46.9% of individuals were involved in half‐sib relationships, even between years, suggesting that source populations were recurrent year after year. There were few individuals resampled between years (30 in 2015 and 14 in 2018), indicating a rapid turnover. Considering the large number of half‐sib relationships but the low number of relations per individual, we conclude that *P. nobilis* exhibit homogeneous reproductive success. Self‐recruitment was not detected, making this population highly vulnerable as replenishment only relies on connectivity from neighboring populations. In the context of the pandemic caused by *H. pinnae*, these results will have to be considered when choosing a location to reintroduce individuals in potential future rescue plans.

## Graphical abstract

This study documents the family structure of a pelagic spawner, *Pinna nobilis*, endemic of the Mediterranean Sea, which is facing a major crisis that threatens its survival as most of its populations have been decimated by a parasite, *Haplosporidium pinnae*. Based on the survey of a wild population during several years and using parentage analyses, we focused on recruitment processes which drive the replenishment of the local population. These results will have serious implications in management plans, particularly in the context of the present pandemic and considering that a number of rescue projects, that include reintroduction perspectives, are emerging in several Mediterranean countries.

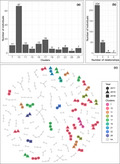

## 1 INTRODUCTION

Coastal organisms, from macroalgae to invertebrates, and even some fish species, are mostly sedentary or have limited movement capabilities as adults and are, for most of them, characterized by a two‐phase life cycle (Cowen & Sponaugle, 2009; Thorson, 1950; Young, 1990). Adults are generally confined to a restricted living area, or fixed, whereas early life stages include a pelagic larval phase that allows for potential long‐distance dispersal (Selkoe et al., 2006). This phase is a fundamental ecological step for coastal organisms, especially in fragmented habitats, as it has a direct impact on local survival through replenishment (Saenz‐Agudelo et al., 2011), maintains a high level of genetic diversity (Almany et al., 2009), controls local distribution and abundances for species, and determines their persistence and resilience in case of local disturbance (Cowen & Sponaugle, 2009; Selkoe et al., 2006).

While understanding dispersal patterns is a major topic in conservation biology, the study of larval dispersal remains a challenge because, due to their small size and high mortality rate, larvae are almost impossible to track. Considering their weak swimming abilities, larvae have been treated as passive particles carried over large distances by sea currents and larval dispersal has been studied mostly through hydrodynamic models (review in Swearer et al., 2019). The dispersal distance of a species has been therefore considered to be proportional to the duration of the larval phase. However, with the emergence of new molecular tools, it is now possible to determine population structure and to estimate connectivity patterns using genetic analyses and statistics (Marko & Hart, 2018). Several authors have shown that high proportions of larvae do not disperse over large distances but stay close to their birth place, and this is the case for various taxa (Bell et al., 2014; Carreras‐Carbonell et al., 2007; Teske et al., 2016; Weersing & Toonen, 2009). More recently, parentage analyses have been shown to be a powerful tool to directly assess dispersal patterns and to understand maintaining processes as the reconstruction of family structure provides information about settlement strategies and the population's contribution to its own replenishment (Berumen et al., 2012; Christie et al., 2010; D'Aloia et al., 2013; Díaz‐Viloria et al., 2013; Dubé et al., 2020; Jones et al., 2005; Planes et al., 2009; Salles et al., 2016; Wilson & Ferguson, 2002). However, family structure is poorly documented for pelagic spawners as parentage analyses require a significant investment in the sampling effort (Jones et al., 2010; Pemberton, 2008).

Having a proportion of self‐recruitment (i.e., the proportion of recruits that settle in their natal population) can have multiple benefits for the population. First, staying at proximity to the parental population can limit the risks linked to long‐distance dispersal. For species which lives in a fragmented habitat, the chance of finding a suitable settlement habitat may also decrease whether larvae disperse too far from the parental population (Swearer et al., 2002).

The fan mussel, *Pinna nobilis* (Linnaeus, 1758), is an endemic bivalve of the Mediterranean Sea that lives in coastal and lagoonal seagrass meadows leading to a patchy and aggregative distribution (Basso et al., 2015). It is a successive hermaphrodite with both male and female gonads active but with staggered developmental stages to avoid self‐fertilization (De Gaulejac, 1995). Gametogenesis occurs from March to June, followed by a succession of alternate gamete emissions and rapid gametogenesis from June to August (De Gaulejac et al., 1995). It is generally accepted that fan mussels are pelagic spawners but their reproduction cycle still remains unclear as Trigos et al. (2018) reported observations of internal fertilization events with females maintaining eggs in their body cavities. After fertilization, pelagic eggs are transformed into pelagic veliger larvae. For *P*. *nobilis*, larval phase duration is around 5–10 days during which larvae are dispersed by marine currents (Butler et al., 1993) but, as for many bivalve species, this stage is poorly documented and a recent study has shown that the larval stage could last up to 20 days under controlled conditions (Trigos et al., 2018). At the end of the pelagic larval stage, individuals that find an appropriate substratum undergo a metamorphosis and settle in soft‐bottom habitats to grow rapidly until they are of adult size (Butler et al., 1993). As for most of the bivalve species, settlement rates can be very inconstant in space and time, with occurrence of exceptional years, as often described in species with very high fertility, that will be critical for the species population dynamics (Beukema et al., 2001; González‐Wangüemert et al., 2014; Miyawaki & Sekiguchi, 2000).

Today, *P*. *nobilis* is facing a major crisis that threatens its survival as most of the populations, throughout the Mediterranean Sea, have suffered mass mortality events (Vázquez‐Luis et al., 2017) caused by a protozoan parasite, *Haplosporidium pinnae* (Catanese et al., 2018). This is an unprecedented situation, whether in terms of mortality rates or spread speed, as most of the affected populations were decimated within about three to four weeks after the detection of the first symptom of infection by *H*. *pinnae* (Cabanellas‐Reboredo et al., 2019; García‐March, Tena, et al., 2020; Šarić et al., 2020). Following this tragic situation, the status of the species was reevaluated and upgraded to “critically endangered” on the IUCN red list (Kersting et al., 2019). Currently, only very few populations remain unaffected in marine habitats (Acarli et al., 2020; Çinar et al., 2021; García‐March, Tena, et al., 2020) or in lagoons (Cabanellas‐Reboredo et al., 2019; Catanese et al., 2018; Peyran, Morage, et al., 2021) that should be considered as targets for conservation priorities as they could act as refuges and source populations for the species recovery.

In this context, it is now, more than ever, necessary to have better information about the demographic dynamics of the species in order to implement effective conservation plans. Previous studies on *P*. *nobilis* population genetics showed low genetic differentiation that implied connectivity among populations (González‐Wangüemert et al., 2019; Peyran, Boissin, et al., 2021; Wesselmann et al., 2018). However, none of these studies focused on recruitment processes which drive the replenishment of the local population. This study documents the functioning of a natural population of a pelagic spawner species, *P*. *nobilis*, by (i) estimating the relative contribution of self‐recruitment vs. recruitment from distant populations on the maintaining of the local population and (ii) establishing family structure to understand settlement strategies of larvae and variability in reproductive success within local populations.

## 2 MATERIALS AND METHODS

### 2.1 Study location and sample collection

The study was conducted in the bay of Peyrefite (42°27.6167′N; 3°9.4833E), which is located in the Cerbère‐Banyuls Marine Protected Area, France, in the Gulf of Lion (north‐western Mediterranean Sea). The bay is shallow, with a mean depth around 4.6 m, and hosts a *P*. *oceanica* meadow of approximately 13,251 m^2^ including dead matte and 5731 m^2^ of live meadow that has the potential to be colonized by *P*. *nobilis* (Iwankow, 2015).

**FIGURE 1:**
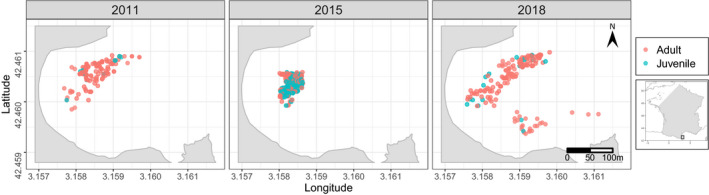
Location of all the *Pinna nobilis* individuals sampled in the bay of Peyrefite during the three fieldwork campaigns

Fan mussel biopsies were collected by SCUBA diving during three fieldwork campaigns in 2011, 2015, and 2018 (see Figure 1). In 2011, the sampling effort was focused on the northern part of the bay, in 2015 on the eastern part, and in 2018, the entire bay was surveyed. In 2018, the fieldwork started at the same time the first signs of the epidemic caused by *H*. *pinnae* were starting to show, and thus, some individuals were already infected by the parasite. In order to carry out the most exhaustive possible sampling, the following strategy was established: fixed baselines, with known GPS positions, were installed and, every 2 m on these baselines, 60‐m line transects where deployed following a heading. The meadow was then meticulously explored along 2 m wide transects. For each fan mussel, a biopsy of ~1 cm^3^ of mantle tissue was collected with forceps. This method was shown to be non‐lethal after an initial test and survey, but as a preventive measure, the smallest individuals were not sampled to avoid potential lethal biopsies. Each tissue sample was stored in absolute ethanol at room temperature before DNA extraction. As GPS positions of the baselines, heading and length of transects were known, GPS positions for each fan mussel were deducted using triangulation. The objective was to sample as many fan mussels as possible but it was not possible for some individuals as they closed their shell when divers approached. However, all individuals in the bay were reported, leading to a precise estimation of the population size for 2018. In 2015, population size was estimated in a previous work (Peyran, Morage, et al., 2021), but in 2011, it was not possible to estimate it as only the GPS positions of biopsied fan mussels were recorded and the surface of the meadow, at this time, was unknown.

### 2.2 Size and sexual maturity stage estimation

As fan mussels live half‐buried in the sediment, it is not possible to directly measure their size, and as such, it must be estimated. The maximum width, the minimum width, and the unburied length were measured *in situ* for each individual, as described by García‐March et al. (2002), and the total length was determined using the equation developed by De Gaulejac (1993).

Size of individuals was then used to estimate their developmental stage related to sexual maturity (i.e., separating immature juveniles from adults that can reproduce) following the method described by Iwankow (2015) where growth models from different studied sites, derived from Martínez et al. (2014), were averaged. The size of sexual maturity was deducted from this mean model and matches to the slow‐down of the growth which is reached at 24.5 cm. Individuals below this threshold were considered as juveniles, and above it, as adults.

### 2.3 Molecular analysis

Genomic DNA was extracted using the QIAxtractor and QIAcube robots (Qiagen) following the manufacturer's instructions. Samples were screened at 30 microsatellite markers from Peyran et al. (2020) and González‐Wangüemert et al. (2014) following the same genotyping procedure as described in Peyran, Boissin, et al. (2021). Allele sizes were scored and checked manually using GeneMapper v.5 software (Applied Biosystems). Samples presenting ambiguous peak profiles were re‐amplified, genotyped, and re‐scored, and all peaks that remained unclear were considered as missing data.

### 2.4 Data analysis

As mollusks are known to often present amplification troubles leading to high frequencies of null alleles (Hare et al., 1996), the quality of the microsatellite markers was checked using MICROCHECKER v2.2.3. (Van Oosterhout et al., 2004). On the original set of 30 markers, 8 presented high frequencies of null alleles and were removed from the database to avoid any bias in further analyses. The final set thus contained 22 polymorphic loci.

Since the sampling areas overlapped between three fieldwork campaigns, some individuals were sampled multiple times, leading to duplicate genotypes in the database. genalex 6.503 (Peakall & Smouse, 2012) was used to identify identical genotypes that corresponded to the same individuals. Both genotypes from the same individual were kept for analyses when comparing genetic composition between years while a unique genotype was kept for parentage analysis.

Genetic diversity was evaluated through allele number (*N*_a_), expected (*H*_e_), unbiased expected (*Hnb*), and observed (*H*_o_) heterozygosities using genalex 6.503 (Peakall & Smouse, 2012). As sample size is not homogeneous among years, standardized allelic richness (*Ar*) and standardized private allelic richness (*Apr*) were estimated using ADZE software (Szpiech et al., 2008), based on a standardized sample size of 150, large enough to be representative of the total sampling. Genetix (Belkhir et al., 2004) was used to compute inbreeding coefficients (*F*_IS_) for each year, using the method of Weir and Cockerham (1984) and the significance of values was estimated by permutations (1 000 permutations per population). Genetix was also used to calculate differentiation index (*F*_ST_) between years using the Robertson and Hill unbiased estimator for *F*_ST_ (Robertson & Hill, 1984) corrected by Raufaste and Bonhomme (2000), RH′. The sequential Bonferroni correction for multiple tests was then applied to adjust *p*‐values (Rice, 1989).

After removing replicate genotypes, a parentage analysis was performed using colony package which implemented a full‐pedigree likelihood method to simultaneously infer sib‐ships and parentage among individuals, considering the entire pedigree configuration rather than pairs of individuals (Jones & Wang, 2010). colony’s algorithm thus allows for individuals to be partitioned, into family clusters, even though they are not directly related *via* their parents (i.e., half‐ or full‐sib) but instead with more complex relations, which is often the case in polygamous population. Individuals within a given cluster are related to at least one other individual from the same cluster, with a full‐sib, half‐sib, or parent–offspring relation. Individuals from different clusters are unrelated. colony provides a likelihood for the entire cluster configuration as well as for each dyad.

In the present study, all individuals were considered as potential offspring, as they could all potentially have a parent present in the bay of Peyrefite, providing thus a dataset of 596 individuals. The potential parent pool included all individuals except for the juveniles sampled in 2018, providing a dataset of 578 potential parents. *P*. *nobilis* is a successive hermaphrodite for which a succession of alternate spawning and fast gametogenesis occurs during the spawning period (De Gaulejac et al., 1995). All parental genotypes were thus considered as candidate fathers and mothers within the analysis since the sex of individuals could not be determined. colony analysis was performed with the following parameters for the three long‐length runs: both sexes are polygamous, the organism is dioecious, and, as it is an external spawner, inbreeding was considered as possible but clonal reproduction was not. The full‐likelihood method was used with a high likelihood precision and no sib‐ships prior. The genotype error rate was set to 1% for all loci. Only family clusters with a probability greater than 95% and inferred relations with a probability also greater than 95% were considered in the results. The resulting relations between individuals were visualized using R package *ggraph* (Pedersen et al., 2017).

## 3 RESULTS

### 3.1 Population size, sampling effort, and recaptures

Even if some individuals may be missed during the fieldwork campaign, the size of the population was around 698 fan mussels in 2018 (Table 1). The total number of sampled individuals was 640, with 224 in 2011, 256 in 2015, which represents 40.5% of the total population and 160 in 2018, representing 22.9% of the population (see Table 1). Among the 640 samples, 44 were recaptures (i.e., a sample of an individual that was already sampled during a previous campaign). In 2015, although there was little overlap between both sampling areas, 30 fan mussels were recaptured from 2011. In 2018, for the 160 individuals sampled, only 11 were previously sampled in 2015 and 3 in 2011, whereas the sampling area covered the entire bay in 2018 and thus totally overlapped both sampling areas of 2011 and 2015. In 2011 and 2018, the samples were dominated by adults as there were only 20 juveniles with 188 adults in 2011 and 18 juveniles with 138 adults in 2018. In 2015, the sampling was mostly composed of young individuals, with 161 juveniles and 93 adults sampled.

**TABLE 1:** Sampling effort for each year, total number of *Pinna nobilis* juveniles, adults, and individuals that were not measured (NA), population size for each fieldwork campaign and proportion of the population that was sampled

	2011	2015	2018	Totals				Population size	Proportion of sampled population
				Juveniles	Adults	NA	Total		
2011	**224**	–	–	20	188	16	224	NA	–
2015	(30)	**226**	–	161	93	2	256	632 ± 256^a^	40.5%
2018	(3)	(11)	**146**	18	138	4	160	698	22.9%

Note

Numbers in bold indicate the number of new individuals sampled during the year and numbers between brackets refer to individuals that were already sampled during the previous years (i.e., recaptures).

^a^ Peyran, Morage, et al. (2021).

### 3.2 Genetic variability among years

The analyses revealed a high level of genetic diversity for the three years as the mean number of alleles per locus (*N*_a_) ranged from 14.773 to 13.591 and the total number of alleles over the 22 loci (*Na*_tot_) ranged from 299 and 325 (see Table 1). The observed (*H*_o_) and expected (*H*_e_) heterozygosities ranged from 0.6845 to 0.7361 and from 0.7121 to 0.7331, respectively. Genetic diversity seemed to be slightly lower in 2018 as *N*_a_ and *Na*_tot_ were lower and only five private alleles (*Nap*) were found in 2018 compared with 20 and 23 for 2011 and 2015. However, this appeared to be due to a lower sampling effort in 2018 as all of the parameters were very similar among years when parameters were estimated for a standardized sample size of 150 individuals (see *Ar*, *Apr*, and *Hnb*; Table 2). Genetic diversity was thus very homogeneous and constant among years. The inbreeding coefficient (*F*_IS_) was very low and non‐significant in 2011 and increased, but remained weak, and significant (*p*‐value < .001) in 2015 and 2018, indicating heterozygote deficiencies. All of the pairwise *F*_ST_ values were very low (ranging from 0.00196 to 0.00235) but two pairwise comparisons were significant after Bonferroni sequential correction: between 2011 and 2015 and between 2015 and 2018, suggesting that the 2015 sample is significantly different (Table 3).

**TABLE 2:** Summary statistics of genetic diversity indices of *Pinna nobilis* for each year

Year	*N*	Na_tot_	*N*_a_	Ar	Nap	Ap	Apr	*H*_o_	*H*_e_	Hnb	*F*_IS_
2011	224	314	14.273	12.6268	20	0.909	0.830754	0.7361	0.7331	0.7348	−0.00179
2015	254	325	14.773	12.5484	23	1.045	0.892598	0.6845	0.7121	0.7135	0.04079***
2018	159	299	13.591	12.8043	5	0.227	1.10422	0.7026	0.7242	0.7265	0.03306***

Note

*N*, number of individuals sampled; *Na*_tot_, total number of alleles; *N*_a_, mean number of alleles; Ar, standardized allelic richness; *Ap*_tot_, total number of private alleles; *Ap*, mean number of private alleles; *Apr*, standardized private allele richness; *H*_o_, observed heterozygosity; *H*_e_, expected heterozygosity; *Hnb*, unbiased expected heterozygosity and *F*_IS_, inbreeding coefficient. Significant values of *F*_IS_ are indicated with *p‐value < .05; **p‐value < .01; ***p‐value < .001.

**TABLE 3:** *F*_ST_ values of pairwise comparisons (Robertson and Hill estimator for *F*_ST_, 1984 corrected by Raufaste & Bonhomme, 2000) between the 3 years of fieldwork campaigns during which *Pinna nobilis* specimens were collected

*F*_ST_	2015	2018
2011	0.00235*	0.00196
2015		0.00233*

Note

The * indicates significant values after Bonferroni sequential correction.

### 3.3 Family structure

**FIGURE 2:**
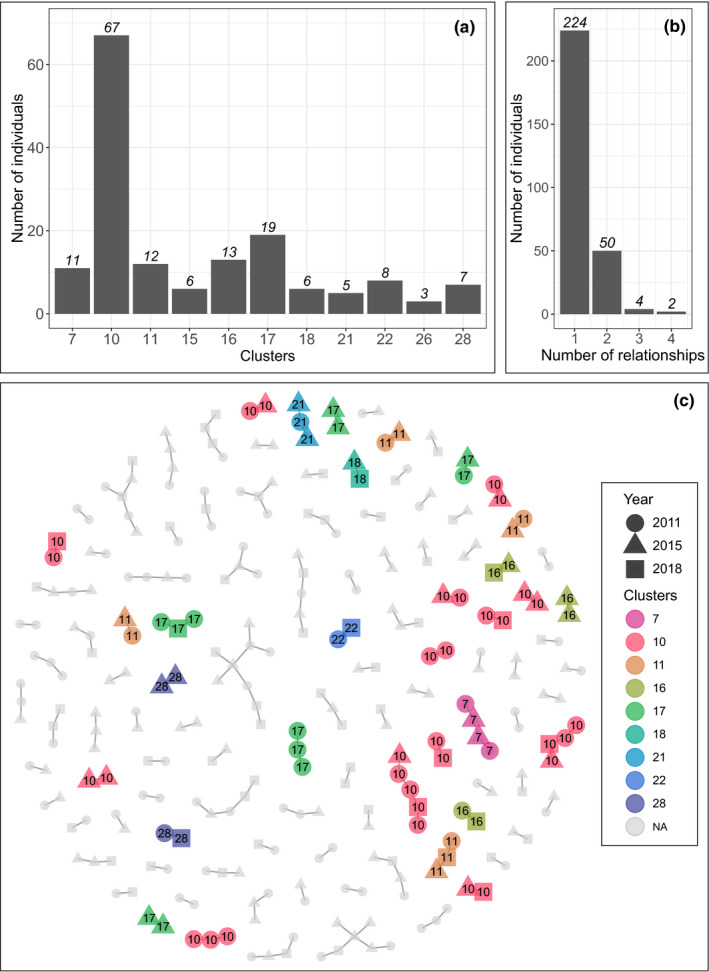
Representation of the family structure highlighted within the fan mussel population in the bay of Peyrefite, based on colony parentage analysis. (a) Number of individuals per family cluster that have at least a 95% probability of grouping together. (b) Number of individuals involved in 1, 2, 3, or 4 half‐sib relationships with a probability up to 95%. (c) Representation of the half‐sib relations with a probability of at least 95% and displayed in color if the cluster of belonging has a probability up to 95% or in gray if the cluster of belonging has a probability lower than 95%. Clusters 15 and 26 are not displayed as all included individuals were linked by relationships with probability lower than 95%

In total, 11 family clusters were found, based on the colony’s parentage analysis when retaining only families with probability higher than 95% (Figure 2a), involving 157 fan mussels. Within these clusters, the number of individuals is 14.3, on average, and ranged from 3 to 67 for Cluster n°10 which is thus largely prevailing. All individuals belonging to a given cluster were related by half‐sib relations (as there was no full‐sib or parent–offspring relation) with at least one individual of the same cluster, but not always with high probabilities (Figure 2c). The clusters were homogeneously constituted of individuals from all years (Table 4, Figure S1). However, the numbers of juveniles and adults were different between years for a given cluster as, for example, Cluster n°10 was composed of 24 adults and only 1 juvenile from 2011 whereas there were 14 juveniles and 8 adults from 2015.

**TABLE 4:** Year of sampling and sexual maturity of the *Pinna nobilis* individuals that constitute the 11 family clusters identified based on the colony’s parentage analysis

Cluster	2011				2015				2018			
	Juveniles	Adults	NA	Total	Juveniles	Adults	NA	Total	Juveniles	Adults	NA	Total
7	0	5	0	5	3	2	0	5	0	1	0	1
10	1	24	1	26	14	8	0	22	2	16	1	19
11	0	5	0	5	5	1	0	6	0	1	0	1
15	0	1	0	1	2	0	0	2	1	2	0	3
16	0	4	0	4	5	2	0	7	1	0	1	2
17	2	6	0	8	7	2	0	9	0	2	0	2
18	0	2	0	2	0	2	0	2	0	2	0	2
21	0	1	0	1	2	1	0	3	0	1	0	1
22	0	2	1	3	2	1	0	3	0	2	0	2
26	0	2	0	2	1	0	0	1	0	0	0	0
28	0	1	0	1	1	3	0	4	0	2	0	2

Over the 596 individuals screened, no parent–offspring or full‐sib relation was detected. However, 280 individuals, that is 46.9% of fan mussels in the study, were implicated in 172 half‐sib relations with a probability of at least 95%. Most of the individuals were involved in only one half‐sib relationship, and only 2 were implicated in 4 relationships (Figure 2b).

## 4 DISCUSSION

This study is among the few to investigate family structure for a wild population of a pelagic spawning species and the first to assess local dispersal and replenishment patterns in a restricted *P*. *nobilis* population. Among the 640 specimens sampled over the 3 years, few were resampled, even in 2018 when the entire bay was surveyed and thus sampling area overlapped those of 2011 and 2015, indicating a quick turnover of individuals in the population. The analyses revealed a high level of genetic diversity in the bay of Peyrefite which is constant over years and the genetic differentiation between years was very low even if significant, with 2015 slightly different from 2011 and 2018, in terms of allele frequencies. The parentage analysis demonstrated that fan mussels are structured in family clusters within the bay, with one family highly dominant, which could suggest a higher reproductive success due to a larger abundance of spawners in the parental population or to a better larval survival rate. Further, almost half of the sampled individuals were related and involved in a half‐sib relationship with another sampled fan mussel, even through the years, indicating that most recruits inside the bay came from the same source population from year to year.

### 4.1 Temporal dynamic within the population

For the 2015 sampling, 30 individuals were previously sampled in 2011, whereas there was little spatial overlap between the two sampling areas. In 2018, while the entire bay was surveyed and sampled as much as possible, there were even less recaptures as only 11 specimens were previously sampled in 2015 and 3 in 2011. Overall, there were very few resampled individuals during the successive fieldwork campaigns, with only 4 and 3 years in between, suggesting that there was a rapid turnover of the population and that in only 7 years, it was almost fully renewed. This result was quite surprising as, even if the population size appeared to be similar between 2015 and 2018, it seems to indicate little survival rate whereas fan mussels are considered to be long‐lived. The life expectancy of fan mussels was previously estimated to be more than 20 years (Butler et al., 1993; García‐March, Hernandis, et al., 2020), and in Rouanet et al. (2015), authors surveyed two individuals for about 45 years. In the literature, authors reported stable populations, with slow dynamics and low mortality rates (in Columbretes Islands, Kersting & García‐March, 2017; in Alfacs Bay, Prado et al., 2014). In Lake Vouliagmeni, Katsanevakis (2007) also reported high mortality as, in a 2‐month period, at a depth of 4 m, 80% of individuals with widths up to 18 cm died. This mortality was attributed to illegal fishing in shallow waters, but it is an unlikely hypothesis in our study as the bay of Peyrefite is located in a marine protected area. According to García‐March et al. (2007) and García‐March, Hernandis, et al. (2020), exposed open sea and shallow habitats, such as the bay of Peyrefite, can be highly stressful for fan mussels as moderate‐to‐high hydrodynamics and waves create disturbances and increase mortality rate. In addition, the area is known to undergo intense air–sea interactions leading to strong winds and large sea states that can have substantial impacts on coastal habitats (Renault et al., 2012), which could be responsible for the lower survival in the bay of Peyrefite.

As a parallel, low but significant genetic differentiation was found between years in the bay of Peyrefite, which also supports the hypothesis of a rapid turnover within the population. Even if low, values were in the same order of magnitude as what was found between Port‐St‐Louis and the Côte des Albères, which are separated by approximately 200 km (Peyran, Boissin, et al., 2021). Such differences, as well as the small heterozygote deficiency observed in 2015 and 2018, may reflect some temporal Wahlund effect that could be due to successive cohorts presenting slight variations in allele frequencies. Genetic differences between cohorts can be caused by different selective histories during the dispersal phase, a change in population source or in the respective contributions of adults with a better reproductive success in the population source. Similar phenomena were documented for reef fish (Pusack et al., 2014) and flatfish (Hoarau et al., 2002), and changes in cohort composition were attributed to variations in reproductive success and local variations in water currents. The large variance in individual reproductive success is well documented. Hedgecock and Pudovkin (2011) formulated the hypothesis of sweepstakes reproductive success, explaining that the probability of matching reproductive activity with oceanographic conditions favorable for gamete maturation, fertilization, larval development, and recruitment are very low and similar to a random draw. The effective size and the cohort genetic composition are thus widely different than that of the whole population.

### 4.2 Reproductive system and maintenance of the population

Although *P*. *nobilis* is usually considered as an external fertilizer and successive hermaphrodite, its reproductive system is still poorly understood as authors reported the occurrence of internal fertilization with females retaining eggs in their body cavity (Trigos et al., 2018) or high rates of self‐fertilization with individuals that were simultaneously male and female (Prado et al., 2020). Estimations of kinship provided here, within a natural population, offer new insights into the reproductive mode of the species. The analysis revealed no full‐sib relationship whereas almost half (46.9%) of the population was genetically linked by a half‐sib relationship. In parallel, individuals were involved in a maximum of 4 relationships meaning that there were a large number of adults involved in the reproduction and that reproductive success is low and homogeneous among spawners. A high level of multiple mating is not surprising for a species with external fertilization and with aggregative behavior, where both males and females release their gametes into the water column. Similar levels of multiple mating were also found for other organisms such as in *Millepora* hydrocorals (Dubé et al., 2020), the colonial ascidian *Botryllus schlosseri* (Johnson & Yund, 2007), the small bivalve *Digitaria digitaria* (Marina et al., 2020), the sea urchin *Abatus agassizii* (Maturana et al., 2017), and other invertebrates (Bishop & Pemberton, 1997; Levitan, 2018; Wacker et al., 2018). Our results support the hypothesis of a pelagic spawner with synchronized spawning events involving a large number of individuals. This is consistent with a previous study that highlighted a high genetic diversity and genetic homogeneity throughout the entire Occitan coastline, even in fragmented and isolated habitats, that could be due to a large effective population size that maintains high polymorphism (Peyran, Boissin, et al., 2021). In the *P*. *nobilis* reproductive cycle, several successive spawning events occur (De Gaulejac et al., 1995), which can also increase the multiple mating as the release of gametes at different times decreases the probability that all of a female's oocytes were fertilized by a single male. This mechanism insures a high level of genetic diversity among offspring but also reduces variation in their fitness that was proven to, slightly but significantly, increase fitness across generations (Fox & Rauter, 2003).

No parent–offspring dyad was detected within the bay of Peyrefite, suggesting that there was no self‐recruitment and that replenishment of the population only depends on larval supply from populations outside the bay. Then, as there were a lot of half‐sib relationships both within and between years, it seems that larvae clouds were not totally scattered during the dispersal phase and rather tended to travel and settle partially together. As individuals are related between years, the parents may be the same year after year, suggesting that the source population may also be similar through years. This finding is consistent with previous studies on *P*. *nobilis* population structure which suggested high levels of connectivity across the Gulf of Lion (González‐Wangüemert et al., 2019; Peyran, Boissin, et al., 2021; Wesselmann et al., 2018). In Wesselmann et al. (2018), the authors revealed genetic differentiations between Banyuls‐Murcia and Banyuls‐Mallorca likely due to isolation by distance and to the North Balearic Front which creates a physical barrier of dense water that limits the circulation of particles such as larvae (Seyfried et al., 2019). The source population of individuals in the bay of Peyrefite was thus more likely located in the Eastern or Northeastern part of the Gulf of Lion, such as the northern lagoons (Salses‐Leucate and Thau), and larvae may be transported by the influence of the Northern Current (Seyfried et al., 2019). Such low levels of self‐recruitment and high connectivity were also found at a similar spatial scale for other bivalve species such as the Arctic surfclam, *Mactromeris polynyma* (Cassista & Hart, 2007), the Venus clam *Polititapes rhomboides* (Chacón et al., 2021), or the hard clam *Meretrix meretrix* (Ye et al., 2020).

### 4.3 Family structure

Most of the individuals were involved in only one or two half‐sib relationships, indicating that parents only generated one or two offspring that survived, demonstrating a reproductive success that is fairly homogeneous among adults in the source population. However, one family (Cluster n°10) was much more abundant than the others, over the three years sampled, and thus had a higher contribution to the replenishment of the population in the bay of Peyrefite. Several scenarios could explain the predominance of this family within the bay. First, the prevalence of offspring from this family could simply be due to a larger abundance of adults from the same family in the source population, which thus proportionately contribute more to the production of larvae than the rest of the population. The number of larvae that recruit in the bay of Peyrefite would thus be larger, even with the same rate of survival than larvae from other families. The dominance of offspring from Cluster n°10 could also be explained by a higher reproductive success of the progenitors. Several parameters can enhance the reproductive success of individuals such as a higher fecundity of fertilization which leads to a small number of progenitors that produce more larvae in the source population, which is a widespread phenomenon for marine invertebrates (Hedgecock et al., 2007; Lallias et al., 2010). However, if offspring were produced by a small number of progenitors, we would have expected more relatives, especially full‐sibs. The dominance of offspring from Cluster 10 could also be due to higher survival of larvae during the dispersal phase. At this stage, we do not have any information about the source populations, the parents, or the initial abundance of larvae, and it is thus not possible to assess the most likely scenario.

### 4.4 Implication for conservation

Our study emphasized a classic family structure for a species with large simultaneous spawning events, aggregative behavior, and a large population size that led to a high level of genetic diversity. Although one family seemed dominant, in terms of abundance, the reproductive success was weak but homogeneous among adults in the source population. There was no self‐recruitment, indicating that the population of the bay of Peyrefite is clearly not self‐sustaining and is highly reliant on the sustainability and reproductive success of surrounding populations. These results will have serious implications in management plans, particularly in the context of the present pandemic, caused by *H*. *pinnae*, and considering that a number of rescue projects, that include reintroduction perspectives, are emerging in several Mediterranean countries.

As *P*. *nobilis* is an external spawner, population density will be an important parameter to consider to maintain a stable population and to enhance reproductive success. Several studies showed that one of the major determinants of fertilization success is the concentration of gametes in the water column (André & Lindegarth, 1995; Levitan, 1991, 2004). As this concentration is quickly diluted after release, it is important that the population density is high enough to ensure proximity between individuals. Crimaldi (2012) also demonstrated that when two mates are less than a meter away from one another, the fertilization success is greater than 50%. Because of the pandemic, fan mussel populations were decimated but few individuals survived and appeared likely to be resistant to the parasite (Prado et al., 2021; Vázquez‐Luis et al., 2021). However, populations are now so scattered that it will be almost impossible for individuals to reproduce, if nothing is done to improve this situation.

There was no self‐recruitment in the fan mussel population in the bay of Peyrefite, meaning that all settled individuals were immigrants from a source population and that all of the locally produced larvae were exported to other locations. This high level of connectivity could be an argument in support of the efficiency of the Cerbère‐Banyuls Marine Protected Area because one of the main benefits of protected areas is their seeding of adjacent areas (Russ & Alcala, 1996). However, to ensure the stability of a population, it is necessary to have a balance between self‐recruitment and external larval supply (Armsworth, 2002; Hastings & Botsford, 2006), which is not the case here. Although the population in the bay of Peyrefite is located in a marine protected area, its replenishment and resilience in response to local disturbance is highly uncertain as it depends on both the persistence of neighboring populations and the larval exportation from these populations. This study thus emphasizes the importance of marine protected area networks where protected areas are connected through larval dispersal, which thus enhances resilience (Berumen et al., 2012; Grorud‐Colvert et al., 2014; Planes et al., 2009; Thiault et al., 2019).

When considering reintroduction projects for conservation, the bay of Peyrefite would appear as the perfect site to re‐establish a population of fan mussels thanks to the dense Posidonia meadows and the protection provided by the marine protected area. Since the extinction of the local population in 2018, no recruitment was observed in this area, indicating that the source populations were likely decimated. Reintroducing a population into the bay of Peyrefite must therefore be carefully thought out as it presents both advantages and disadvantages. In the one hand, reinstalling a prolific population in the bay would lead to the potential exportation of larvae and contribute to the repopulation of other sites. However, on the other hand, the becoming of larvae produced within the population is uncertain and there is no evidence that they will actually settle in neighboring populations as they may be lost in the open sea or die because of the risks linked to the dispersal phase. Then, the population will have to be regularly replenished by reintroducing new individuals as, beside the low survival rate, the population is not self‐sustaining and there will not be recruitment until the surrounding populations have recovered. This feature will thus have to be considered when choosing a location to reintroduce individuals in potential future rescue plans.

## CONFLICT OF INTEREST

The authors declare that they have no competing interests.

## AUTHOR CONTRIBUTIONS

**Claire Peyran:** Formal analysis (equal); Writing – original draft (equal). **Emilie Boissin:** Writing – review & editing (equal). **Titouan Morage:** Resources (equal). **Elisabet Nebot‐Colomer:** Resources (equal). **Guillaume Iwankow:** Resources (equal). **Serge Planes:** Conceptualization (equal); Funding acquisition (equal); Project administration (equal); Supervision (equal); Writing – review & editing (equal).

## Supporting information

Supplementary Material

## Data Availability

The microsatellite genotypes for all analyzed individuals are available on Dryad.org: https://doi.org/10.5061/dryad.g79cnp5r4.

## References

[ece38482-bib-0001] Acarli, S. , Acarli, D. , & Kale, S. (2020). Current status of critically endangered Fan Mussel *Pinna nobilis* (Linnaeus 1758) Population in Çanakkale Strait, Turkey. Marine Science and Technology Bulletin, 10, 62–70. 10.33714/masteb.793885

[ece38482-bib-0002] Almany, G. R. , Connolly, S. R. , Heath, D. D. , Hogan, J. D. , Jones, G. P. , McCook, L. J. , Mills, M. , Pressey, R. L. , & Williamson, D. H. (2009). Connectivity, biodiversity conservation and the design of marine reserve networks for coral reefs. Coral Reefs, 28, 339–351. 10.1007/s00338-009-0484-x

[ece38482-bib-0003] André, C. , & Lindegarth, M. (1995). Fertilization efficiency and gamete viability of a sessile, free‐spawning bivalve, *Cerastoderma edule* . Ophelia, 43, 215–227. 10.1080/00785326.1995.10429833

[ece38482-bib-0004] Armsworth, P. R. (2002). Recruitment limitation, population regulation, and larval connectivity in Reef Fish metapopulations. Ecology, 83, 1092. 10.2307/3071916

[ece38482-bib-0005] Basso, L. , Hendriks, I. , Steckbauer, A. , & Duarte, C. (2015). Resistance of juveniles of the Mediterranean pen shell, (*Pinna nobilis*) to hypoxia and interaction with warming. Estuarine, Coastal and Shelf Science, 165, 199–203. 10.1016/j.ecss.2015.05.016

[ece38482-bib-0006] Belkhir, K. , Borsa, P. , Chikhi, L. , Raufaste, N. , & Bonhomme, F. (2004). GENETIX 4.05, Population genetics software for Windows TM. Univ. Montpellier II.

[ece38482-bib-0007] Bell, J. J. , Smith, D. , Hannan, D. , Haris, A. , Jompa, J. , & Thomas, L. (2014). Resilience to disturbance despite limited dispersal and self‐recruitment in tropical barrel sponges: Implications for conservation and management. PLoS One, 9, e91635. 10.1371/journal.pone.0091635 24651687 PMC3961256

[ece38482-bib-0008] Berumen, M. L. , Almany, G. R. , Planes, S. , Jones, G. P. , Saenz‐Agudelo, P. , & Thorrold, S. R. (2012). Persistence of self‐recruitment and patterns of larval connectivity in a marine protected area network. Ecology and Evolution, 2, 444–452. 10.1002/ece3.208 22423335 PMC3298954

[ece38482-bib-0009] Beukema, J. , Dekker, R. , Essink, K. , & Michaelis, H. (2001). Synchronized reproductive success in the main bivalve species in the Wadden Sea: Causes and consequences. Marine Ecology Progress Series, 211, 143–155. 10.3354/meps211143

[ece38482-bib-0010] Bishop, J. D. , & Pemberton, A. J. (1997). Sessile animals: Attached, but promiscuous? Trends in Ecology & Evolution, 12, 403. 10.1016/S0169-5347(97)87390-9 21238130

[ece38482-bib-0011] Butler, A. , Vicente, N. , & de Gaulejac, B. (1993). Ecology of the pterioid bivalves *Pinna bicolor* Gmelin and *Pinna nobilis* L. Marine Life, 3(1‐2), 37–45.

[ece38482-bib-0012] Cabanellas‐Reboredo, M. , Vázquez‐Luis, M. , Mourre, B. , Álvarez, E. , Deudero, S. , Amores, Á. , Addis, P. , Ballesteros, E. , Barrajón, A. , Coppa, S. , García‐March, J. R. , Giacobbe, S. , Casalduero, F. G. , Hadjioannou, L. , Jiménez‐Gutiérrez, S. V. , Katsanevakis, S. , Kersting, D. , Mačić, V. , Mavrič, B. , … Hendriks, I. E. (2019). Tracking a mass mortality outbreak of pen shell *Pinna nobilis* populations: A collaborative effort of scientists and citizens. Scientific Reports, 9, 13355. 10.1038/s41598-019-49808-4 31527825 PMC6746856

[ece38482-bib-0013] Carreras‐Carbonell, J. , Macpherson, E. , & Pascual, M. (2007). High self‐recruitment levels in a Mediterranean littoral fish population revealed by microsatellite markers. Marine Biology, 151, 719–727. 10.1007/s00227-006-0513-z

[ece38482-bib-0014] Cassista, M. C. , & Hart, M. W. (2007). Spatial and temporal genetic homogeneity in the Arctic surfclam (*Mactromeris polynyma*). Marine Biology, 152, 569–579. 10.1007/s00227-007-0711-3

[ece38482-bib-0015] Catanese, G. , Grau, A. , Valencia, J. M. , Garcia‐March, J. R. , Vázquez‐Luis, M. , Alvarez, E. , Deudero, S. , Darriba, S. , Carballal, M. J. , & Villalba, A. (2018). *Haplosporidium pinnae sp. nov*., a haplosporidan parasite associated with mass mortalities of the fan mussel, Pinna nobilis, in the Western Mediterranean Sea. Journal of Invertebrate Pathology, 157, 9–24. 10.1016/j.jip.2018.07.006 30005968

[ece38482-bib-0016] Chacón, G. M. , Arias‐Pérez, A. , Freire, R. , Martínez, L. , Ojea, J. , & Insua, A. (2021). Genetic characterization of wild, broodstock and seed samples of *Polititapes rhomboides* (Bivalvia: *Veneridae*): Implications for hatchery seed production. Aquaculture Reports, 20, 100658. 10.1016/j.aqrep.2021.100658

[ece38482-bib-0017] Christie, M. R. , Tissot, B. N. , Albins, M. A. , Beets, J. P. , Jia, Y. , Ortiz, D. M. , Thompson, S. E. , & Hixon, M. A. (2010). Larval connectivity in an effective network of marine protected areas. PLoS One, 5, e15715. 10.1371/journal.pone.0015715 21203576 PMC3006342

[ece38482-bib-0018] Çinar, M. E. , Bilecenoğlu, M. , Yokeş, M. B. , & Güçlüsoy, H. ( 2021). *Pinna nobilis* in the south Marmara Islands (Sea of Marmara); it still remains uninfected by the epidemic and act s as egg laying substratum for an alien invader. Mediterranean Marine Science, 22(1), 161–168. 10.12681/mms.25289

[ece38482-bib-0019] Cowen, R. K. , & Sponaugle, S. (2009). Larval Dispersal and marine population connectivity. Annual Review of Marine Science, 1, 443–466. 10.1146/annurev.marine.010908.163757 21141044

[ece38482-bib-0020] Crimaldi, J. P. (2012). The role of structured stirring and mixing on gamete dispersal and aggregation in broadcast spawning. Journal of Experimental Biology, 215, 1031–1039. 10.1242/jeb.060145 22357596

[ece38482-bib-0021] D'Aloia, C. C. , Bogdanowicz, S. M. , Majoris, J. E. , Harrison, R. G. , & Buston, P. M. (2013). Self‐recruitment in a Caribbean reef fish: A method for approximating dispersal kernels accounting for seascape. Molecular Ecology, 22, 2563–2572. 10.1111/mec.12274 23495725

[ece38482-bib-0022] De Gaulejac, B. (1993). Etude écophysiologique du mollusque bivalve méditerranéen *Pinna nobilis* L. reproduction; croissance; respiration.

[ece38482-bib-0023] De Gaulejac, B. (1995). Mise en évidence de l’hermaphrodisme successif à maturation asynchrone de *Pinna nobilis* . Comptes Rendus De L'académie Des Sciences, 318, 99–103.

[ece38482-bib-0024] De Gaulejac, B. , Henry, M. , & Vicente, N. (1995). An ultrastructural study of gametogenesis of the marine bivalve *Pinna nobilis* (Linnaeus 1758) II. Spermatogenesis. Journal of Molluscan Studies, 61, 393–403. 10.1093/mollus/61.3.393

[ece38482-bib-0025] Díaz‐Viloria, N. , Próo, S.‐A.‐G.‐G.‐D. , Cruz, P. , & Perez‐Enriquez, R. (2013). Assessment of self‐recruitment in a pink abalone (*Haliotis corrugata*) aggregation by parentage analyses. Journal of Shellfish Research, 32, 105–113. 10.2983/035.032.0116

[ece38482-bib-0026] Dubé, C. E. , Boissin, E. , Mercière, A. , & Planes, S. (2020). Parentage analyses identify local dispersal events and sibling aggregations in a natural population of *Millepora* hydrocorals, a free‐spawning marine invertebrate. Molecular Ecology, 29, 1508–1522. 10.1111/mec.15418 32227655

[ece38482-bib-0027] Fox, C. W. , & Rauter, C. M. (2003). Bet‐hedging and the evolution of multiple mating. Evolutionary Ecology Research, 5, 273–286.

[ece38482-bib-0028] García‐March, J. R. , García‐Carrascosa, A. M. , & Pena, Á. L. (2002). In situ measurement of *Pinna nobilis* shells for age and growth studies: A new device. Marine Ecology, 23, 207–217. 10.1046/j.1439-0485.2002.02781.x

[ece38482-bib-0029] García‐March, J. R. , Hernandis, S. , Vázquez‐Luis, M. , Prado, P. , Deudero, S. , Vicente, N. , & Tena‐Medialdea, J. (2020). Age and growth of the endangered fan mussel *Pinna nobilis* in the western Mediterranean Sea. Marine Environment Research, 153, 104795. 10.1016/j.marenvres.2019.104795 31587816

[ece38482-bib-0030] García‐March, J. R. , Pérez‐Rojas, L. , & García‐Carrascosa, A. M. (2007). Influence of hydrodynamic forces on population structure of Pinna nobilis L., 1758 (Mollusca: Bivalvia): The critical combination of drag force, water depth, shell size and orientation. Journal of Experimental Marine Biology and Ecology, 342, 202–212. 10.1016/j.jembe.2006.09.007

[ece38482-bib-0031] García‐March, J. R. , Tena, J. , Henandis, S. , Vázquez‐Luis, M. , López, D. , Téllez, C. , Prado, P. , Navas, J. I. , Bernal, J. , Catanese, G. , Grau, A. , López‐Sanmartín, M. , Nebot‐Colomer, E. , Ortega, A. , Planes, S. , Kersting, D. , Jimenez, S. , Hendriks, I. , Moreno, D. , … Deudero, S. (2020). Can we save a marine species affected by a highly infective, highly lethal, waterborne disease from extinction? Biological Conservation, 243, 108498. 10.1016/j.biocon.2020.108498

[ece38482-bib-0032] González‐Wangüemert, M. , Basso, L. , Balau, A. , Costa, J. , Renault, L. , Serrão, E. A. , Duarte, C. M. , & Hendriks, I. E. (2019). Gene pool and connectivity patterns of *Pinna nobilis* in the Balearic Islands (Spain, Western Mediterranean Sea): Implications for its conservation through restocking. Aquatic Conservation: Marine and Freshwater Ecosystems, 29, 175–188. 10.1002/aqc.2976

[ece38482-bib-0033] González‐Wangüemert, M. , Costa, J. , Basso, L. , Duarte, C. , Serrão, E. , & Hendriks, I. (2014). Highly polymorphic microsatellite markers for the Mediterranean endemic fan mussel *Pinna nobilis* . Mediterranean Marine Science, 16, 31. 10.12681/mms.949

[ece38482-bib-0035] Grorud‐Colvert, K. , Claudet, J. , Tissot, B. N. , Caselle, J. E. , Carr, M. H. , Day, J. C. , Friedlander, A. M. , Lester, S. E. , de Loma, T. L. , Malone, D. , & Walsh, W. J. (2014). Marine protected area networks: Assessing whether the whole is greater than the sum of its parts. PLoS One, 9, e102298. 10.1371/journal.pone.0102298 25084458 PMC4118840

[ece38482-bib-0036] Hare, M. P. , Karl, S. A. , & Avise, J. C. (1996). Anonymous nuclear DNA markers in the American oyster and their implications for the heterozygote deficiency phenomenon in marine bivalves. Molecular Biology and Evolution, 13, 334–345. 10.1093/oxfordjournals.molbev.a025593 8587499

[ece38482-bib-0037] Hastings, A. , & Botsford, L. W. (2006). Persistence of spatial populations depends on returning home. Proceedings of the National Academy of Sciences of the United States of America, 103, 6067–6072. 10.1073/pnas.0506651103 16608913 PMC1458697

[ece38482-bib-0038] Hedgecock, D. , Launey, S. , Pudovkin, A. I. , Naciri, Y. , Lapègue, S. , & Bonhomme, F. (2007). Small effective number of parents (*Nb*) inferred for a naturally spawned cohort of juvenile European flat oysters *Ostrea edulis* . Marine Biology, 150, 1173–1182. 10.1007/s00227-006-0441-y

[ece38482-bib-0039] Hedgecock, D. , & Pudovkin, A. I. (2011). Sweepstakes reproductive success in highly fecund marine fish and shellfish: A review and commentary. Bulletin of Marine Science, 87, 971–1002. 10.5343/bms.2010.1051

[ece38482-bib-0040] Hoarau, G. , Rijnsdorp, A. D. , Van Der Veer, H. W. , Stam, W. T. , & Olsen, J. L. (2002). Population structure of plaice (*Pleuronectes platessa* L.) in northern Europe: Microsatellites revealed large‐scale spatial and temporal homogeneity. Molecular Ecology, 11, 1165–1176. 10.1046/j.1365-294X.2002.01515.x 12074724

[ece38482-bib-0041] Iwankow, G. Influence des herbiers de posidonies (*Posidonia oceanica*) sur la distribution des grandes nacres de Méditerranée (*Pinna nobilis*) le long de la côte rocheuse des Albères. Ecole Pratique des Hautes Etudes (112 pp).

[ece38482-bib-0042] Johnson, S. L. , & Yund, P. O. (2007). Variation in multiple paternity in natural populations of a free‐spawning marine invertebrate. Molecular Ecology, 16, 3253–3262. 10.1111/j.1365-294X.2007.03366.x 17651201

[ece38482-bib-0043] Jones, A. G. , Small, C. M. , Paczolt, K. A. , & Ratterman, N. L. (2010). A practical guide to methods of parentage analysis. Molecular Ecology Resources, 10, 6–30. 10.1111/j.1755-0998.2009.02778.x 21564987

[ece38482-bib-0044] Jones, G. P. , Planes, S. , & Thorrold, S. R. (2005). Coral reef fish larvae settle close to home. Current Biology, 15, 1314–1318. 10.1016/j.cub.2005.06.061 16051176

[ece38482-bib-0045] Jones, O. R. , & Wang, J. (2010). COLONY: A program for parentage and sibship inference from multilocus genotype data. Molecular Ecology Resources, 10, 551–555. 10.1111/j.1755-0998.2009.02787.x 21565056

[ece38482-bib-0046] Katsanevakis, S. (2007). Density surface modelling with line transect sampling as a tool for abundance estimation of marine benthic species: The *Pinna nobilis* example in a marine lake. Marine Biology, 152, 77–85. 10.1007/s00227-007-0659-3

[ece38482-bib-0047] Kersting, D. K. , & García‐March, J. R. (2017). Long‐term assessment of recruitment, early stages and population dynamics of the endangered Mediterranean fan mussel *Pinna nobilis* in the Columbretes Islands (NW Mediterranean). Marine Environment Research, 130, 282–292. 10.1016/j.marenvres.2017.08.007 28870538

[ece38482-bib-0048] Kersting, D. , Mouloud, B. , Cizmek, H. , Grau, A. , Jimenez, C. , Katsanevakis, S. , Oztürk, B. , Tuncer, S. , Tunesi, L. , Vázquez‐Luis, M. , Vicente, N. , & Otero, M. (2019). *Pinna nobilis*. The IUCN Red List of Threatened Species, 2019. 10.2305/IUCN.UK.2019-3.RLTS.T160075998A160081499.en

[ece38482-bib-0049] Lallias, D. , Taris, N. , Boudry, P. , Bonhomme, F. , & Lapègue, S. (2010). Variance in the reproductive success of flat oyster *Ostrea edulis* L. assessed by parentage analyses in natural and experimental conditions. Genetics Research, 92, 175–187. 10.1017/S0016672310000248 20667162

[ece38482-bib-0050] Levitan, D. R. (1991). Influence of body size and population density on fertilization success and reproductive output in a free‐spawning invertebrate. Biological Bulletin, 181, 261–268. 10.2307/1542097 29304646

[ece38482-bib-0051] Levitan, D. R. (2004). Density‐dependent sexual selection in external fertilizers: Variances in male and female fertilization success along the continuum from sperm limitation to sexual conflict in the Sea Urchin *Strongylocentrotus franciscanus* . American Naturalist, 164, 298–309. 10.1086/423150 15478086

[ece38482-bib-0052] Levitan, D. R. (2018). Do sperm really compete and do eggs ever have a choice? Adult distribution and gamete mixing influence sexual selection, sexual conflict, and the evolution of gamete recognition proteins in the sea. American Naturalist, 191, 88–105. 10.1086/694780 29244565

[ece38482-bib-0053] Marina, P. , Urra, J. , Bueno, J. D. D. , Rueda, J. L. , Gofas, S. , & Salas, C. (2020). Spermcast mating with release of zygotes in the small dioecious bivalve *Digitaria digitaria* . Scientific Reports, 10, 12605. 10.1038/s41598-020-69457-2 32724126 PMC7387346

[ece38482-bib-0054] Marko, P. B. , & Hart, M. W. (2018). Genetic analysis of larval dispersal, gene flow, and connectivity, evolutionary ecology of marine invertebrate larvae. Oxford University Press.

[ece38482-bib-0055] Martínez, A. , Trigos, S. , García‐march, J. , Vicente, N. , Torres, J. , Deudero, S. , Toro, M. , & Tena, J. (2014). Comparative study of growth of the endangered bivalve *Pinna nobilis* in marine protected areas vs. unprotected areas of the western Mediterranean Sea. XVIII Simp. Ibérico Estud. Biol. Mar.

[ece38482-bib-0056] Maturana, C. S. , Gérard, K. , Díaz, A. , David, B. , Féral, J.‐P. , & Poulin, E. (2017). Mating system and evidence of multiple paternity in the Antarctic brooding sea urchin *Abatus agassizii* . Polar Biology, 40, 787–797. 10.1007/s00300-016-2001-3

[ece38482-bib-0057] Miyawaki, D. , & Sekiguchi, H. (2000). Long‐Term observations on larval recruitment processes of bivalve assemblages on temperate tidal flats. Benthos Research, 55, 1–16. 10.5179/benthos1996.55.1_1

[ece38482-bib-0058] Peakall, R. , & Smouse, P. E. (2012). GenAlEx 6.5: Genetic analysis in Excel. Population genetic software for teaching and research – An update. Bioinformatics, 28, 2537–2539. 10.1093/bioinformatics/bts460 22820204 PMC3463245

[ece38482-bib-0059] Pedersen, T. L. , Pedersen, M. T. L. , LazyData, T. , Rcpp, I. , & Rcpp, L. (2017). Package ‘ggraph’.

[ece38482-bib-0060] Pemberton, J. (2008). Wild pedigrees: The way forward. Proceedings of the Royal Society B: Biological Sciences, 275, 613–621. 10.1098/rspb.2007.1531 PMC2386891 18211868

[ece38482-bib-0061] Peyran, C. , Boissin, E. , Morage, T. , Nebot‐Colomer, E. , Iwankow, G. , & Planes, S. (2021). Genetic homogeneity of the critically endangered fan mussel, *Pinna nobilis*, throughout lagoons of the Gulf of Lion (North‐Western Mediterranean Sea). Scientific Reports, 11, 7805. 10.1038/s41598-021-87493-4 33833376 PMC8032772

[ece38482-bib-0062] Peyran, C. , Morage, T. , Nebot‐Colomer, E. , Iwankow, G. , & Planes, S. (2021). Unexpected residual habitats raise hope for the survival of the fan mussel, *Pinna nobilis*, along the Occitan coast (north‐western Mediterranean Sea), Manuscript submitted for publication.

[ece38482-bib-0063] Peyran, C. , Planes, S. , Tolou, N. , Iwankow, G. , & Boissin, E. (2020). Development of 26 highly polymorphic microsatellite markers for the highly endangered fan mussel *Pinna nobilis* and cross‐species amplification. Molecular Biology Reports, 47(4), 2551–2559. 10.1007/s11033-020-05338-1 32095986

[ece38482-bib-0064] Planes, S. , Jones, G. P. , & Thorrold, S. R. (2009). Larval dispersal connects fish populations in a network of marine protected areas. Proceedings of the National Academy of Sciences of the United States of America, 106, 5693–5697. 10.1073/pnas.0808007106 19307588 PMC2659712

[ece38482-bib-0065] Prado, P. , Andree, K. B. , Trigos, S. , Carrasco, N. , Caiola, N. , García‐March, J. R. , Tena, J. , Fernández‐Tejedor, M. , & Carella, F. (2020). Breeding, planktonic and settlement factors shape recruitment patterns of one of the last remaining major population of *Pinna nobilis* within Spanish waters. Hydrobiologia, 847, 771–786. 10.1007/s10750-019-04137-5

[ece38482-bib-0066] Prado, P. , Caiola, N. , & Ibáñez, C. (2014). Habitat use by a large population of *Pinna nobilis* in shallow waters. Scientia Marina, 78, 555–565. 10.3989/scimar.04087.03A

[ece38482-bib-0067] Prado, P. , Grau, A. , Catanese, G. , Cabanes, P. , Carella, F. , Fernández‐Tejedor, M. , Andree, K. B. , Añón, T. , Hernandis, S. , Tena, J. , & García‐March, J. R. (2021). *Pinna nobilis* in suboptimal environments are more tolerant to disease but more vulnerable to severe weather phenomena. Marine Environment Research, 163, 105220. 10.1016/j.marenvres.2020.105220 33302153

[ece38482-bib-0068] Pusack, T. J. , Christie, M. R. , Johnson, D. W. , Stallings, C. D. , & Hixon, M. A. (2014). Spatial and temporal patterns of larval dispersal in a coral‐reef fish metapopulation: Evidence of variable reproductive success. Molecular Ecology, 23, 3396–3408. 10.1111/mec.12824 24917250

[ece38482-bib-0069] Raufaste, N. , & Bonhomme, F. (2000). Properties of bias and variance of two multiallelic estimators of FST. Theoretical Population Biology, 57, 285–296. 10.1006/tpbi.2000.1457 10828220

[ece38482-bib-0070] Renault, L. , Chiggiato, J. , Warner, J. C. , Gomez, M. , Vizoso, G. , & Tintoré, J. (2012). Coupled atmosphere‐ocean‐wave simulations of a storm event over the Gulf of Lion and Balearic Sea. Journal of Geophysical Research: Oceans, 117, 1–25. 10.1029/2012JC007924

[ece38482-bib-0071] Rice, W. R. (1989). Analyzing tables of statistical tests. Evolution, 43, 223–225. 10.1111/j.1558-5646.1989.tb04220.x 28568501

[ece38482-bib-0072] Robertson, A. , & Hill, W. G. (1984). Deviations from Hardy‐Weinberg proportions: Sampling variances and use in estimation of inbreeding coefficients. Genetics, 107, 703–718. 10.1093/genetics/107.4.703 6745643 PMC1202385

[ece38482-bib-0073] Rouanet, E. , Trigos, S. , & Vicente, N. (2015). From youth to death of old age: The 50‐year story of a *Pinna nobilis* fan mussel population at Port‐Cros Island (Port‐Cros National Park, Provence, Mediterranean Sea). 209‐Scientific Reports of the Port‐Cros National Park, 29, 209–222.

[ece38482-bib-0074] Russ, G. R. , & Alcala, C. A. (1996). Do marine reserves export adult fish biomass? Evidence from Apo Island, Central Philippines. Marine Ecology Progress Series, 132, 1–9.

[ece38482-bib-0075] Saenz‐Agudelo, P. , Jones, G. P. , Thorrold, S. R. , & Planes, S. (2011). Connectivity dominates larval replenishment in a coastal reef fish metapopulation. Proceedings of the Royal Society B: Biological Sciences, 278, 2954–2961. 10.1098/rspb.2010.2780 PMC3151711 21325328

[ece38482-bib-0076] Salles, O. C. , Pujol, B. , Maynard, J. A. , Almany, G. R. , Berumen, M. L. , Jones, G. P. , Saenz‐Agudelo, P. , Srinivasan, M. , Thorrold, S. R. , & Planes, S. (2016). First genealogy for a wild marine fish population reveals multigenerational philopatry. Proceedings of the National Academy of Sciences of the United States of America, 113, 13245–13250. 10.1073/pnas.1611797113 27799530 PMC5135361

[ece38482-bib-0077] Šarić, T. , Župan, I. , Aceto, S. , Villari, G. , Palić, D. , De Vico, G. , & Carella, F. (2020). Epidemiology of Noble Pen Shell (*Pinna nobilis* L. 1758) mass mortality events in Adriatic Sea is characterised with rapid spreading and acute disease progression. Pathogens, 9(10), 776. 10.3390/pathogens9100776 PMC7598175 32977433

[ece38482-bib-0078] Selkoe, K. A. , Gaines, S. D. , Caselle, J. E. , & Warner, R. R. (2006). Current shifts and kin aggregation explain genetic patchiness in fish recruits. Ecology, 87, 3082–3094. 10.1890/0012-9658(2006)87#;3082:csakae#;2.0.co;2 17249233

[ece38482-bib-0079] Seyfried, L. , Estournel, C. , Marsaleix, P. , & Richard, E. (2019). Dynamics of the North Balearic Front during an autumn tramontane and mistral storm: Air–sea coupling processes and stratification budget diagnostic. Ocean Science, 15, 179–198. 10.5194/os-15-179-2019

[ece38482-bib-0080] Swearer, S. E. , Shima, J. S. , Hellberg, M. E. , Thorrold, S. R. , Jones, G. P. , Robertson, D. R. , Morgan, S. G. , Selkoe, K. A. , Ruiz, G. M. , & Warner, R. R. (2002). Evidence of self‐recruitment in demersal marine populations. Bulletin of Marine Science, 70, 251–271.

[ece38482-bib-0081] Swearer, S. E. , Treml, E. A. , & Shima, J. S. (2019). A review of biophysical models of marine larval dispersal. In S. J. Hawkins , A. L. Allcock , A. E. Bates , L. B. Firth , I. P. Smith , S. E. Swearer , & P. A. Todd (Eds.), Oceanography and marine biology: An annual review. 57, (pp. 325–356). Taylor & Francis.

[ece38482-bib-0082] Szpiech, Z. A. , Jakobsson, M. , & Rosenberg, N. A. (2008). ADZE: A rarefaction approach for counting alleles private to combinations of populations. Bioinformatics, 24, 2498–2504. 10.1093/bioinformatics/btn478 18779233 PMC2732282

[ece38482-bib-0083] Teske, P. R. , Sandoval‐Castillo, J. , van Sebille, E. , Waters, J. , & Beheregaray, L. B. (2016). Oceanography promotes self‐recruitment in a planktonic larval disperser. Scientific Reports, 6, 34205. 10.1038/srep34205 27687507 PMC5043232

[ece38482-bib-0084] Thiault, L. , Kernaléguen, L. , Osenberg, C. W. , Lison de Loma, T. , Chancerelle, Y. , Siu, G. , & Claudet, J. (2019). Ecological evaluation of a marine protected area network: A progressive‐change BACIPS approach. Ecosphere, 10, e02576. 10.1002/ecs2.2576

[ece38482-bib-0085] Thorson, G. (1950). Reproductive and larval ecology of marine bottom invertebrates. Biological Reviews, 25, 1–45. 10.1111/j.1469-185X.1950.tb00585.x 24537188

[ece38482-bib-0086] Trigos, S. , Vicente, N. , Prado, P. , & Espinós, F. J. (2018). Adult spawning and early larval development of the endangered bivalve *Pinna nobilis* . Aquaculture, 483, 102–110. 10.1016/j.aquaculture.2017.10.015

[ece38482-bib-0087] Van Oosterhout, C. , Hutchinson, W. F. , Wills, D. P. M. , & Shipley, P. (2004). micro‐checker: Software for identifying and correcting genotyping errors in microsatellite data. Molecular Ecology Notes, 4, 535–538. 10.1111/j.1471-8286.2004.00684.x

[ece38482-bib-0088] Vázquez‐Luis, M. , Álvarez, E. , Barrajón, A. , García‐March, J. R. , Grau, A. , Hendriks, I. E. , Jiménez, S. , Kersting, D. , Moreno, D. , Pérez, M. , Ruiz, J. M. , Sánchez, J. , Villalba, A. , & Deudero, S. (2017). S.O.S. *Pinna nobilis*: A mass mortality event in Western Mediterranean Sea. Frontiers in Marine Science, 4, 1–6. 10.3389/fmars.2017.00220

[ece38482-bib-0089] Vázquez‐Luis, M. , Nebot‐Colomer, E. , Deudero, S. , Planes, S. , & Boissin, E. (2021). Natural hybridization between pen shell species: *Pinna rudis* and the critically endangered *Pinna nobilis* may explain parasite resistance in *P. nobilis* . Molecular Biology Reports, 48, 997–1004. 10.1007/s11033-020-06063-5 33394229

[ece38482-bib-0090] Wacker, S. , Larsen, B. M. , Jakobsen, P. , & Karlsson, S. (2018). High levels of multiple paternity in a spermcast mating freshwater mussel. Ecology and Evolution, 8, 8126–8134. 10.1002/ece3.4201 30250689 PMC6145300

[ece38482-bib-0091] Weersing, K. , & Toonen, R. (2009). Population genetics, larval dispersal, and connectivity in marine systems. Marine Ecology Progress Series, 393, 1–12. 10.3354/meps08287

[ece38482-bib-0092] Weir, B. S. , & Cockerham, C. C. (1984). Estimating F‐statistics for the analysis of population structure. Evolution, 38, 1358. 10.2307/2408641 28563791

[ece38482-bib-0093] Wesselmann, M. , González‐Wangüemert, M. , Serrão, E. A. , Engelen, A. H. , Renault, L. , García‐March, J. R. , Duarte, C. M. , & Hendriks, I. E. (2018). Genetic and oceanographic tools reveal high population connectivity and diversity in the endangered pen shell *Pinna nobilis* . Scientific Reports, 8, 4770. 10.1038/s41598-018-23004-2 29555926 PMC5859023

[ece38482-bib-0094] Wilson, A. J. , & Ferguson, M. M. (2002). Molecular pedigree analysis in natural populations of fishes: Approaches, applications, and practical considerations. Canadian Journal of Fisheries and Aquatic Science, 59, 1696–1707. 10.1139/f02-127

[ece38482-bib-0095] Ye, Y. , Yan, C. , Senanan, W. , Guo, B. , Xu, K. , & Lü, Z. (2020). Genetic population structure of the hard clam *Meretrix meretrix* along the Chinese Coastlines revealed by microsatellite DNA markers. Frontiers in Marine Science, 7, 1–11. 10.3389/fmars.2020.00516 32802822

[ece38482-bib-0096] Young, C. M. (1990). Larval ecology of marine invertebrates: A sesquicentennial history. Ophelia, 32, 1–48. 10.1080/00785236.1990.10422023

