# Supplementary material for: Investigating population dynamics from parentage analysis in the highly endangered fan mussel Pinna nobilis
Source: Ecol Evol. 2022 Jan 25;12(1):e8482. doi: 10.1002/ece3.8482 (PMC8796933; doi:10.1002/ece3.8482)
Supplement: Supplementary file 1 — Supplementary Material [file ECE3-12-e8482-s001.docx]

**Supplemental Information for:**

**Investigating population dynamics from parentage analysis in the highly endangered fan mussel Pinna nobilis**

Claire Peyran^1^, Emilie Boissin^1,2^, Titouan Morage^1^, Elisabet Nebot-Colomer^1,3^, Guillaume Iwankow^1^, Serge Planes^1,2^

^1^ PSL Research University: EPHE - UPVD - CNRS, USR 3278 CRIOBE, 66860 Perpignan, France

^2^ Laboratoire d'Excellence « CORAIL »,

^3^ Instituto Español de Oceanografía (IEO, CSIC). Centro Oceanográfico de Baleares. Muelle de Poniente s/n, 07015. Palma de Mallorca, Spain

Corresponding author: [claire.peyran@gmail.com](mailto:claire.peyran@gmail.com)

**
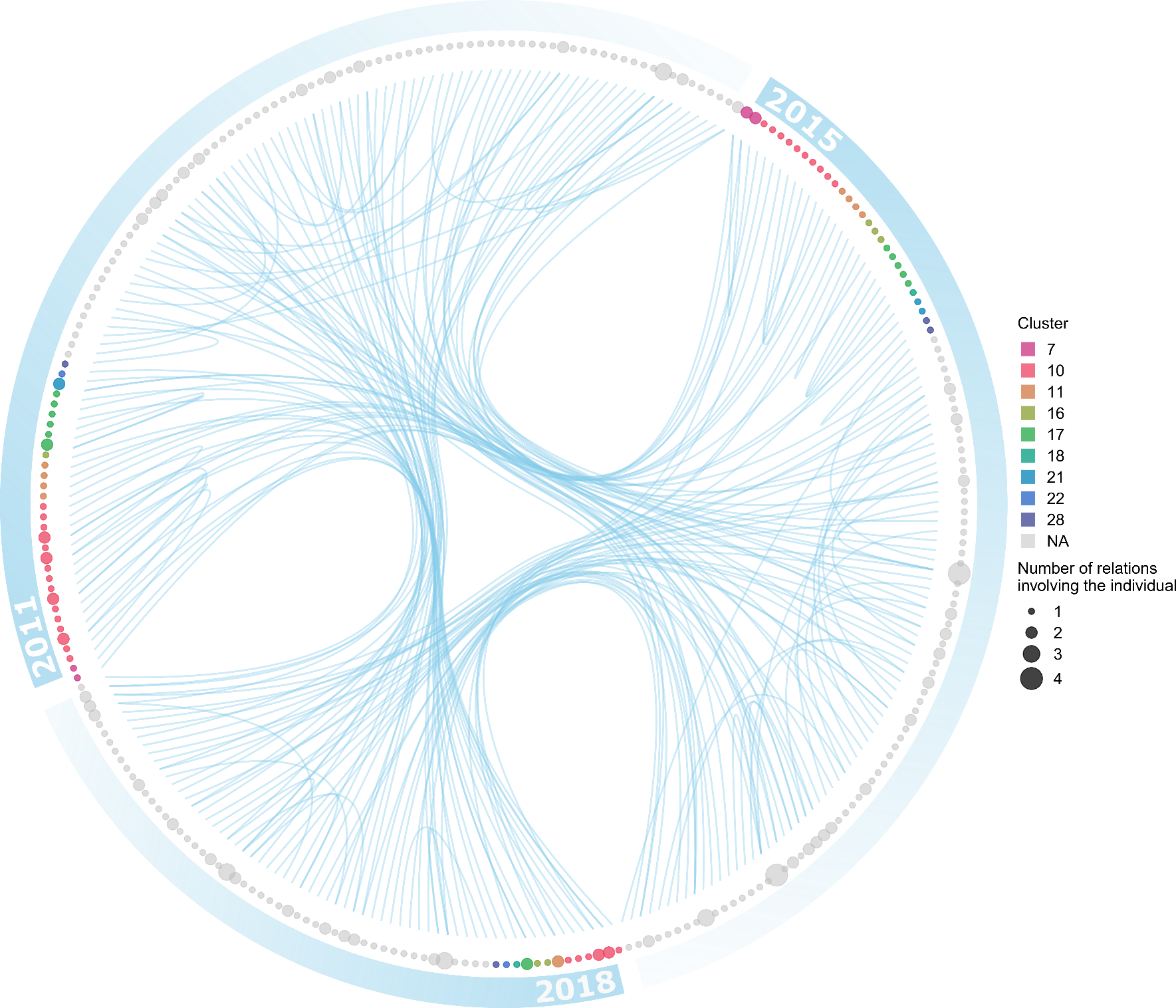
**

Figure S1: Representation of half-sib relations, with probability higher than 95 % that connect Pinna nobilis individuals between years. For each individual the size of the dot depends on the number of relations in which the individual is involved. For each individual the dot is displayed in color if the cluster of belonging has a probability up to 95 % or in grey if the cluster of belonging has a probability lower than 95 %. Clusters 15 and 26 are not displayed as all included individuals were linked by relationships with probability lower than 95 %.
